# Supplementary material for: A new level of RNA-based plant protection: dsRNAs designed from functionally characterized siRNAs highly effective against Cucumber mosaic virus
Source: Nucleic Acids Res. 2025 Mar 19;53(5):gkaf136. doi: 10.1093/nar/gkaf136 (PMC11904787; doi:10.1093/nar/gkaf136)
Supplement: gkaf136_Supplemental_File [file gkaf136_supplemental_file.pdf]

Supplementary data

Supplementary Materials and methods

Analysis of DCL-mediated processing of *e*dsRNA

200 ng ^{32}P-labeled RNA was incubated for up to 24 h at 25°C in 20 µl reactions containing 50% (v/v) BYL and the buffer conditions described before. Total RNA was isolated from the reaction by treatment with proteinase K (Thermo Scientific, #EO0491), followed by chloroform extraction and ethanol precipitation. RNAs were separated on 15% denaturing urea-polyacrylamide gels in TBE buffer and siRNAs were visualized by phosphor-imaging (Typhoon Trio+, GE Healthcare, Chalfont St Giles, UK). To analyze the siRNAs resulting from processing of the *e*dsRNA by RNA-seq, 1.25 µg RNA was incubated for 4 h in a 50 µl reaction containing 50% (v/v) BYL using the aforementioned conditions. Isolation of total RNA and RNA-seq was performed as described above.

Western Blotting

For the immunodetection of *in vitro* synthesized, FLAG-tagged AGO proteins, samples were separated on 10% polyacrylamide gels and western blotting was performed at standard conditions using semi-dry transfer. Polyclonal ANTI-FLAG-tag antibody (Sigma-Aldrich, #F7425-.2MG) was used at a 1:1000 dilution and detected by IRDye 800CW goat anti-rabbit IgG secondary antibody (LI-COR, Lincoln, NE, #926-32211) using a LI-COR ODYSSEY CLx imager.

Silver staining of RNAs

siRNAs that were co-purified in AGO immunoprecipitations were separated on 15% (w/v) denaturing urea-polyacrylamide gels and visualized by silver staining (1). The gel was first fixed in 50% (v/v) methanol, 12% (v/v) acetic acid, and 0.0185% (v/v) formaldehyde for 1 h and subsequently washed three times for 20 min each in 50% (v/v) ethanol. Following pre-treatment with 0.02% (w/v) $Na_2S_2O_3 \times 5\ H_2O$ for 1 min, the gel was rinsed three times for 20 s with water and incubated for 20 min in a solution containing 0.2% (w/v) $AgNO_3$ and 0.028% (v/v) formaldehyde. After rinsing twice with water for 20 s each time, the image development was performed in 6% (w/v) Na_2CO_3, 0.0185% (v/v) formaldehyde, and 0.0004% (w/v) $Na_2S_2O_3 \times 5\ H_2O$ for 10 min. The gel was washed twice with water for 2 min each time and the staining reaction was stopped by incubating for 10 min in a solution of 50% (v/v) methanol and 12% (v/v) acetic acid. Finally, the gel was washed in 50% (v/v) MeOH for at least 20 min.

Monitoring of viral RNA replication

RNA isolated from CMV-inoculated plants was treated with RNase-free DNase I (Roche Diagnostics, #04716728001) and reverse-transcribed using random hexamer primers and RevertAid M-MuLV Reverse Transcriptase (Thermo Scientific, #EP0441) according to the manufacturer's instructions. PCR amplification was performed using DreamTaq DNA Polymerase (Thermo Scientific, #EP0705) and primer pairs that bind in the 3' region of each of the three CMV RNAs (**Supplementary Figure S3**) or in the RNA 2 (**Supplementary Figure S8**).

Supplementary Discussion

Considerations on the different efficiency of 21 and 22 nt long *e*siRNAs and *e*dsRNAs consisting of 21 and 22 nt *e*siRNA sequences

Previous reports have shown that 21 nt siRNAs predominate over 22 nt siRNAs in CMV-infected plants (2–6) and, as in other plant virus infection systems, evidence for a redundant role of DCL4 and DCL2 has been presented (7,8). Further observations indicated that efficient secondary siRNA-dependent silencing of CMV-Δ2b in *A. thaliana* relies on DCL4-dependent 21 nt siRNAs, but not on DCL2-dependent 22 nt siRNAs. The latter were insufficient to confer resistance to the virus in the absence of DCL4 (4). Overall, this suggests that the processing of dsRNAs by DCL4 and the generation of 21 nt siRNAs are particularly important for the induction of an antiviral RNA-silencing response against CMV. Our *in vitro* and *in planta* data support this proposal. They show that 21 nt-long *e*siRNAs and *e*dsRNAs containing 21 nt *e*siRNA sequences are more efficient in the antiviral RNA-silencing process than 22 nt *e*siRNAs and *e*dsRNAs containing 22 nt *e*siRNA sequences (**Figure 6** and **Supplementary Figure S4**). We have no evidence to indicate that the 22 nt *e*siRNAs used in this study enhanced CMV silencing by inducing secondary siRNA production.

Supplementary Tables

Supplementary Table S1. List of oligonucleotides

**DNA oligonucleotides**		
Name	Sequence (5'-3')	Purpose
T7CMV2_MK1f	CCCTAATACGACTCACTATAGGTTTATTTAC AAGAGCGTAC	PCR primer for the generation of template DNA for *in vitro* transcription of CMV RNA 1 and 2, (+)-strand
TraCMV1T_MK1r	TGGTCTCCTTTTAGAGACC	PCR primer for the generation of template DNA for *in vitro* transcription of CMV RNA 1, (+)-strand
TraCMV2T_MK1r	TGGTCTCCTTTTGGAGGCC	PCR primer for the generation of template DNA for *in vitro* transcription of CMV RNA 2 and 3, (+)-strand
T7CMV3_MK1f	CCCTAATACGACTCACTATAGGTAATCTTAC CACTGTGTGTG	PCR primer for the generation of template DNA for *in vitro* transcription of CMV RNA 3, (+)-strand

TraCMV2G_MK1r	GGGTCTCCTTTTGGAGGCC	PCR primer for the generation of template DNA for *in vitro* transcription of CMV RNA 2 and 3, (+)-strand with 3' terminal C
T7CMV2_MK1r	CCCTAATACGACTCACTATAGGGTCTCCTTTTGGAGGCC	PCR primer for the generation of template DNA for *in vitro* transcription of CMV RNA 2 and 3, (-)-strand with 5' terminal G
TraCMV2_MK1f	GGTTTATTTACAAGAGCGTAC	PCR primer for the generation of template DNA for *in vitro* transcription of CMV RNA 2, (-)-strand
TraCMV3_MK1f	GGTAATCTTACCACTGTGTGTG	PCR primer for the generation of template DNA for *in vitro* transcription of CMV RNA 3, (-)-strand
T7VSVdsTra1f	GTCTTTCAGGAAAAAAACTAACAGATATCATGGATATGCTAGGTAATACGACTCACTATAGGGGGTCTTC	oligonucleotide for generating a modified pUC18 vector that contains opposite T7 promoters flanking two BpiI (BbsI) sites
T7VSVdsTra1r	TCGAGAAGACCCCCTATAGTGAGTCGTATTACCTAGCATATCCATGATATCTGTTAGTTTTTTTCCTGAAAGACTGCA	oligonucleotide for generating a modified pUC18 vector that contains opposite T7 promoters flanking two BpiI (BbsI) sites
T7VSVdsTra2f	CTAGGAAGACCCCTATAGTGAGTCGTATTACCTAGCATATCCATGATATCTGTTAGTTTTTTTCCTGAAAGAG	oligonucleotide for generating a modified pUC18 vector that contains opposite T7 promoters flanking two BpiI (BbsI) sites
T7VSVdsTra2r	GATCCTCTTTCAGGAAAAAAACTAACAGATATCATGGATATGCTAGGTAATACGACTCACTATAGGGGTCTTC	oligonucleotide for generating a modified pUC18 vector that contains opposite T7 promoters flanking two BpiI (BbsI) sites
T7VSVadapt1f	TCGAAAGCGGCCGCCCATGGTT	oligonucleotide for generating a modified pUC18 vector that contains opposite T7 promoters flanking two BpiI (BbsI) sites
T7VSVadapt1r	CTAGAACCATGGGCGGCCGCTT	oligonucleotide for generating a modified pUC18 vector that contains opposite T7 promoters flanking two BpiI (BbsI) sites
fragGFP168-1f	GACCATGAAGACTCTAGGGCGAATTGGGTACCGGGCAAGATCTGAGTCCGGACTTGT	PCR primer for cloning of a GFP gene fragment used as template for the generation of an unspecific control dsRNA
fragGFP168-1r	GACCATGAAGACTCATAGGGAGACCGGCAGATCTGATGTCCACACAATCTGCCCTTTC	PCR primer for cloning of a GFP gene fragment used as template for the generation of an unspecific control dsRNA
fragCMV168-1f	GACCATGAAGACTCTAGGGCGAATTGGGTACCGGGGAATAACCGGGTACATCGCGAG	PCR primer for cloning of a CMV RNA 2 cDNA fragment used as template for the generation of a control dsRNA
fragCMV168-1r	GACCATGAAGACTCATAGGGAGACCGGCAGATCTGAACCGAACCATGAAGTGTTTT	PCR primer for cloning of a CMV RNA 2 cDNA fragment used as template for the generation of a control dsRNA
siR6CMV21-1f	TAGGGCGAATTGGGTACCGGGCATTACGTTTCTTAATTGCTGTATACTCTTCTTATGATACGTTATCAGATTTTTCAAGGTAATCT	oligonucleotide for cloning of CMV RNA 2 cDNA fragments used as template for the generation of an edsRNA consisting of six 21 nt esiRNAs
siR6CMV21-2f	TGATGAGCTCCTTGTCGCTTTTTTGTTCGAAGTTCTTACTCTTTCGTCGAAAGTGCAGACTATTCTCAGATCTGCCGGTCTCC	oligonucleotide for cloning of CMV RNA 2 cDNA fragments used as template for the generation of an edsRNA consisting of six 21 nt esiRNAs
siR6CMV21-1r	ATCAAGATTACCTTGAAAAATCTGATAACGTATCATAAGAAGAGTATACAGCAATTAAGAAACGTAATGCCCGGTACCCAATTCGC	oligonucleotide for cloning of CMV RNA 2 cDNA fragments used as template for the generation of an edsRNA consisting of six 21 nt esiRNAs
siR6CMV21-2r	ATAGGGAGACCGGCAGATCTGAGAATAGTCTGCACTTTCGACGAAAGAGTAAGAACTTCGAACAAAAAAGCGACAAGGAGCTC	oligonucleotide for cloning of CMV RNA 2 cDNA fragments used as template for the generation of an edsRNA consisting of six 21 nt esiRNAs
siR6CMV22-1f	TAGGGCGAATTGGGTACCGGGCCATTACGTTTCTTAATTGCTGTAATACTCTTCTTATGATACGTTATTCAGATTTTTCAAGGTAATCTT	oligonucleotide for cloning of CMV RNA 2 cDNA fragments used as template for the generation of an edsRNA consisting of six 22 nt esiRNAs
siR6CMV22-2f	ATGATGAGCTCCTTGTCGCTTTATTTGTTCGAAGTTCTTACTCTGTTCGTCGAAAGTGCAGACTATTCATCAGATCTGCCGGTCTCC	oligonucleotide for cloning of CMV RNA 2 cDNA fragments used as template for the generation of an edsRNA consisting of six 22 nt esiRNAs
siR6CMV22-1r	TCATAAGATTACCTTGAAAAATCTGAATAACGTATCATAAGAAGAGTATTACAGCAATTAAGAAACGTAATGGCCCGGTACCCAATTCGC	oligonucleotide for cloning of CMV RNA 2 cDNA fragments used as template for the generation of an edsRNA consisting of six 22 nt esiRNAs

siR6CMV22-2r	ATAGGGAGACCGGCAGATCTGATGAATAG TCTGCACTTTCGACGAACAGAGTAAGAACT TCGAACAAATAAAGCGACAAGGAGCTCA	oligonucleotide for cloning of CMV RNA 2 cDNA fragments used as template for the generation of an edsRNA consisting of six 22 nt esiRNAs
SeqUPN	AGGGTTTTCCCAGTCACGACGTTG	vector-specific PCR primer for the generation of template DNA for *in vitro* transcription of the top strand of edsRNAs and control dsRNAs
TrasiR6CMV-A1-1r	GGGAGACCGGCAGATCTG	PCR primer for the generation of template DNA for *in vitro* transcription of the top strand of edsRNAs and control dsRNAs with blunt ends
TrasiR6CMV-A1-2r	TAGGGAGACCGGCAGATCTG	PCR primer for the generation of template DNA for *in vitro* transcription of the top strand of edsRNAs and control dsRNAs with 2 nt 3’ overhang
SeqRPN	CAATTTCACACAGGAAACAGCTATG	vector-specific PCR primer for the generation of template DNA for *in vitro* transcription of the bottom strand of edsRNAs and control dsRNAs
TrasiR6CMV-A2-1r	GGGCGAATTGGGTACCGG	PCR primer for the generation of template DNA for *in vitro* transcription of the bottom strand of edsRNAs and control dsRNAs with blunt ends
TrasiR6CMV-A2-2r	TAGGGCGAATTGGGTACCGG	PCR primer for the generation of template DNA for *in vitro* transcription of the bottom strand of edsRNAs and control dsRNAs with 2 nt 3’ overhang
T7_CMV-S_3’reg1r	CCCTAATACGACTCACTATAGGGCACCCGT ACCCTGAAACTAGC	PCR primer for the detection of CMV infection by RT-PCR
Tra_CMV-S_3’reg1f	GGCGGGATCTGAGTTGGC	PCR primer for the detection of CMV infection by RT-PCR
CMV-R2-PCR-1f	AGTACAGAGTTCAGGGTTGAGCG	PCR primer for the detection of CMV infection by RT-PCR
CMV-R2-PCR-1r	CACAAAAGTGGGGGGCACC	PCR primer for the detection of CMV infection by RT-PCR
**RNA oligonucleotides[a]**		
Name	Sequence (5’-3’)	Purpose
siR gf698-21U gs	uaguucauccaugccaugugu	guide strand of siRNA gf698, 5’ U
siR gf698-21U ps	acauggcauggaugaacuaua	passenger strand of siRNA gf698, (5’ U in gs)
siR gf698-21A gs	aaguucauccaugccaugugu	guide strand of siRNA gf698, 5’ A
siR gf698-21A ps	acauggcauggaugaacuuua	passenger strand of siRNA gf698, (5’ A in gs)
siR359-22 gs	ucagauuuuucaagguaaucuu	guide strand of 22 nt variant of siRNA 359
siR359-22 ps	gauuaccuugaaaaaucugaug	passenger strand of 22 nt variant of siRNA 359
siR380-22 gs	aaagcgacaaggagcucaucau	guide strand of 22 nt variant of siRNA 380
siR380-22 ps	gaugagcuccuugucgcuuuug	passenger strand of 22 nt variant of siRNA 380
siR1020-22 gs	auagucugcacuuucgacgaac	guide strand of 22 nt variant of siRNA 1020
siR1020-22 gs	ucgucgaaagugcagacuauuc	passenger strand of 22 nt variant of siRNA 1020
siR1172-22 gs	uuacguuucuuaauugcuguaa	guide strand of 22 nt variant of siRNA 1172
siR1172-22 ps	acagcaauuaagaaacguaaug	passenger strand of 22 nt variant of siRNA 1172
siR1489-22 gs	uacucuucuuaugauacguuau	guide strand of 22 nt variant of siRNA 1489
siR1489-22 ps	aacguaucauaagaagaguaua	passenger strand of 22 nt variant of siRNA 1489
siR2041-22 gs	agaguaagaacuucgaacaaau	guide strand of 22 nt variant of siRNA 2041
siR2041-22 ps	uuguucgaaguucuuacucucu	passenger strand of 22 nt variant of siRNA 2041

[a]Sequences of RNA oligonucleotides that correspond to the 21 nt *e*siRNA candidates targeting CMV RNAs 2 and 3 are listed in tables 1 and 2, respectively

Supplementary Table S2. Accumulation of CMV siRNAs in AGO/RISC

CMV RNA 2					
**AGO1 IP/DCL-processed pool[a]**			**AGO2 IP/DCL-processed pool[a]**		
**siRNA**	**5' nt**	**log2Fold change**	**siRNA**	**5' nt**	**log2Fold change**
2441	U	6.6	2634	A	5.6
2562	U	5.5	1054	A	5.3
186	U	5.4	2041	A	5.0
1613	U	4.9	1020	A	4.8
1844	G	4.7	557	A	4.7
1489	U	4.6	2801	A	4.7
149	U	4.5	2863	A	4.6
1982	U	4.5	2955	A	4.5
2727	U	4.5	1248	A	4.5
1172	U	4.5	380	A	4.5
**AGO1 IP (not detected in DCL-processed pool)[b]**			**AGO2 IP (not detected in DCL-processed pool)[b]**		
**siRNA**	**5' nt**	**mean abundance (%)**	**siRNA**	**5' nt**	**mean abundance (%)**
359	U	0.00608	540	A	0.01022
2740	U	0.00608	449	A	0.00461
			2748	A	0.00403
			407	A	0.00389
**CMV RNA 3**					
**AGO1 IP/DCL-processed pool[a]**			**AGO2 IP/DCL-processed pool[a]**		
**siRNA**	**5' nt**	**log2Fold change**	**siRNA**	**5' nt**	**log2Fold change**
196	A	6.6	667	C	7.4
507	U	5.0	496	A	6.4
34	C	4.7	592	A	5.3
33	A	4.7	593	A	5.3
985	U	4.4	478	A	5.2
239	U	4.1	1569	A	4.9
2061	U	3.8	1019	A	4.9
151	U	3.7	733	A	4.7
2174	U	3.7	35	A	4.5
1098	U	3.7	2099	A	4.3
**AGO1 IP (not detected in DCL-processed pool)[b]**			**AGO2 IP (not detected in DCL-processed pool)[b]**		
**siRNA**	**5' nt**	**mean abundance (%)**	**siRNA**	**5' nt**	**mean abundance (%)**
988	U	0.00642	1394	A	0.00574
			1132	A	0.00463
			358	A	0.00390

[a]siRNAs were sorted according to the ratio (log2Fold change) of their abundance in the AGO immunoprecipitation to their abundance in the initial siRNA pool generated from dsRNAs by BYL-endogenous DCLs.
[b]siRNAs listed here were not detectable in the initial siRNA pool (i.e. a log2Fold change could not be calculated) but showed in the AGO immunoprecipitation a higher abundance than at least one of the siRNAs listed above.

Supplementary Figure S1

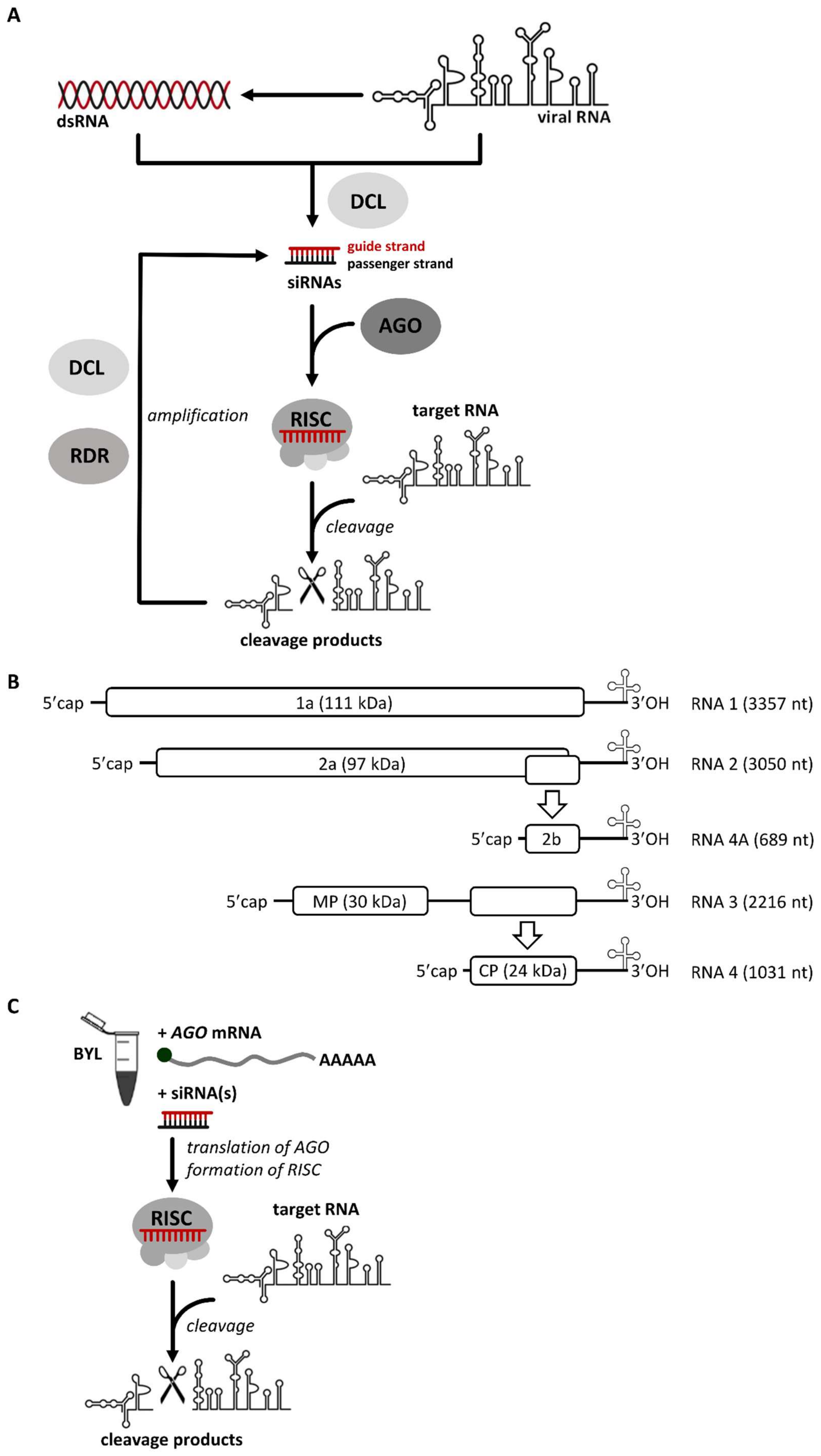

Supplementary Figure S1. Schematic representations of the antiviral RNAi pathway in plants, the genomic organization of CMV and the *in vitro* slicer assay. (A) Antiviral RNAi pathway that leads to the slicing (cleavage) of a viral target RNA (for a detailed description, see text). Viral genomic RNA or double-stranded (ds) versions of the viral RNA such as replication intermediates are processed by DCLs into siRNAs. SiRNA guide strands are incorporated into AGO/RISC, which then may catalyze endonucleolytic cleavage of the complementary target viral RNA. Secondary siRNAs can be generated in an amplification mechanism mediated by the activity of RDRs and DCLs (see text). **(B)** Genomic organization of the CMV genome. All viral RNAs are capped and contain a tRNA-like structure at their 3’ ends. Untranslated regions (UTRs) are shown as lines, ORFs as boxes. The names and molecular weights of the proteins encoded by the genomic (RNAs 1, 2 and 3) and subgenomic RNAs (RNAs 4A and 4) are indicated. MP, movement protein; CP, capsid protein. **(C)** *In vitro* “slicer assay” with BYL that reproduces RISC-mediated slicing (cleavage) of a target RNA with a translated AGO protein of choice. The technical details of the assay are described in the text. It is important to note that the assay can be performed with siRNAs generated endogenously from a dsRNA by the DCLs present in BYL as well as with synthetic siRNAs added exogenously.

Supplementary Figure S2

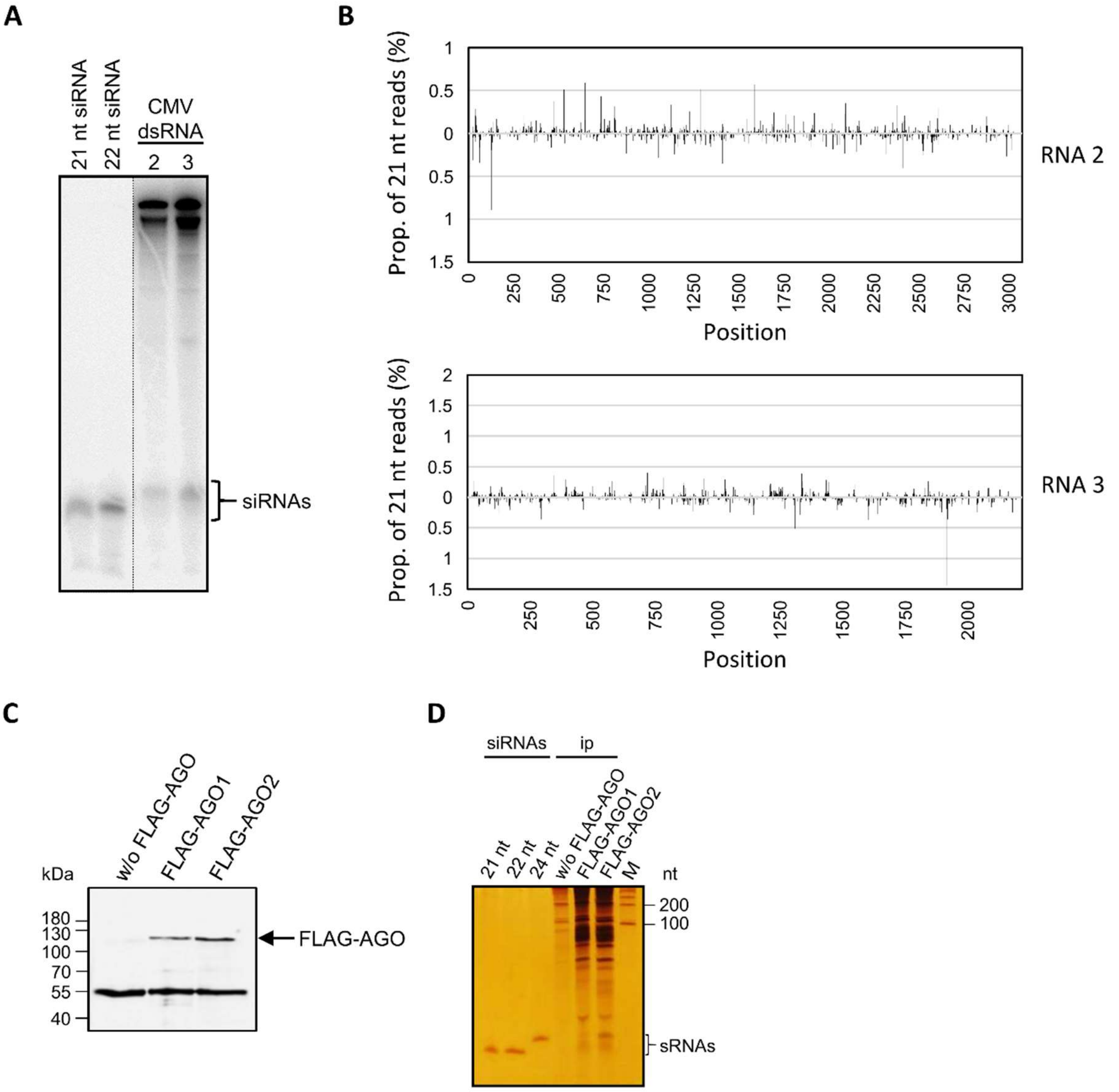

Supplementary Figure S2. DCL-mediated processing of double-stranded versions of CMV RNAs 2 and 3 in BYL and isolation of AGO-bound siRNAs (*e*NA screen steps 1 and 2). (A) Denaturing PAGE and autoradiography of ^{32}P-labeled, double-stranded (ds) versions of CMV RNAs 2 and 3 that were processed in BYL by endogenous DCLs. Synthetic, single-stranded RNAs of a length of 21 and 22 nt served as markers. **(B)** Distribution and abundance of 21 nt siRNAs that were processed by BYL-endogenous DCLs from ds versions of CMV RNAs 2 and 3: total RNA was isolated and the small RNA fraction analyzed by RNA-seq. Peaks above and below the axis represent siRNAs derived from viral (+) strand RNA and viral (-) strand RNA, respectively. Peaks correspond to the position of the 5' nucleotide of the respective siRNA strands and indicate the proportion of all 21 nt reads that map to RNA 2 or RNA 3. Data represent the mean values from three experiments. **(C)** Exemplary western blot of FLAG-AGO samples collected after stringent washing of the ANTI-FLAG M2 affinity gel during the course of

the immunoprecipitation procedure (see Supplementary Materials and methods). The precipitated FLAG-AGO proteins are indicated. A non-specific protein band of 55 kDa was obtained in all immunoprecipitation tests. It presumably corresponds to the heavy ANTI-FLAG IgG-chain, which was also immune-stained. **(D)** Detection of AGO-bound small RNAs. RNAs were isolated from immunoprecipitated FLAG-AGO proteins, separated by denaturing PAGE, and visualized by silver staining. Synthetic, single-stranded siRNAs of 21 nt, 22 nt, and 24 nt, respectively, were used for comparison. M = RNA molecular weight marker. The increased background obtained with the immunoprecipitated AGOs was explained by cellular RNA molecules associated non-specifically to the proteins.

Supplementary Figure S3

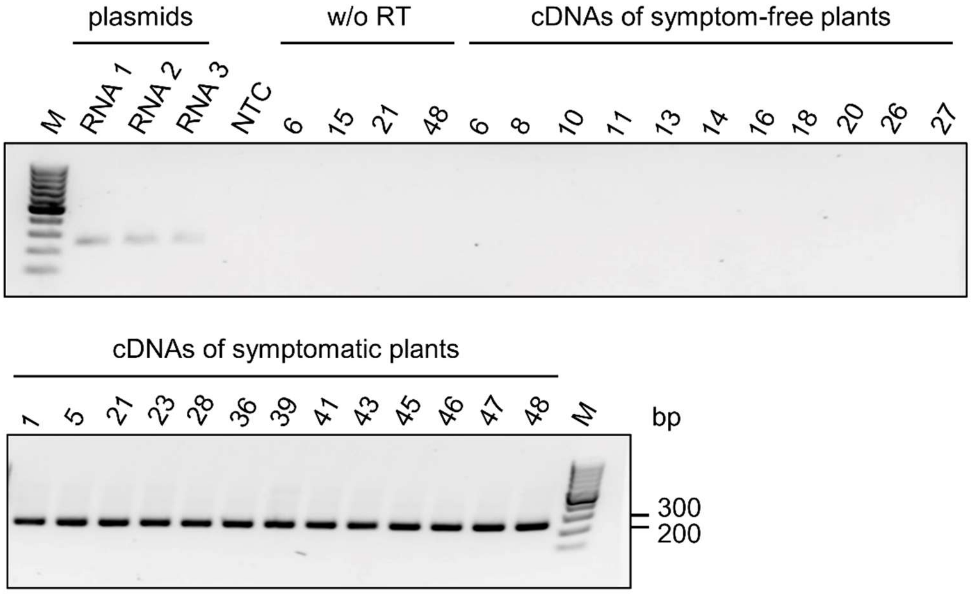

Supplementary Figure S3. Detection of CMV infection by RT-PCR. Leaf discs of asymptomatic and symptomatic *N. benthamiana* plants treated with siRNAs and genomic CMV RNAs (see text) were collected at 35 dpi. The numbers correspond to the numbering of the respective plants from the infection experiment performed with siRNAs directed against CMV RNA 2 (**Figure 3**). Total RNA was extracted and cDNA synthesized using Reverse Transcriptase (RT). This was followed by PCR to detect a conserved sequence in CMV RNAs 1, 2 and 3 and separation of the PCR products on an agarose gel. Amplification of the CMV-specific sequence from plasmids containing the cDNA sequence of the respective viral RNA served as positive control. Samples without addition of RT to the cDNA synthesis reaction (w/o RT) and a "no template control" (NTC, addition of water instead of cDNA during the PCR reaction) served as negative controls (M = GeneRuler 100 bp DNA ladder).

Supplementary Figure S4

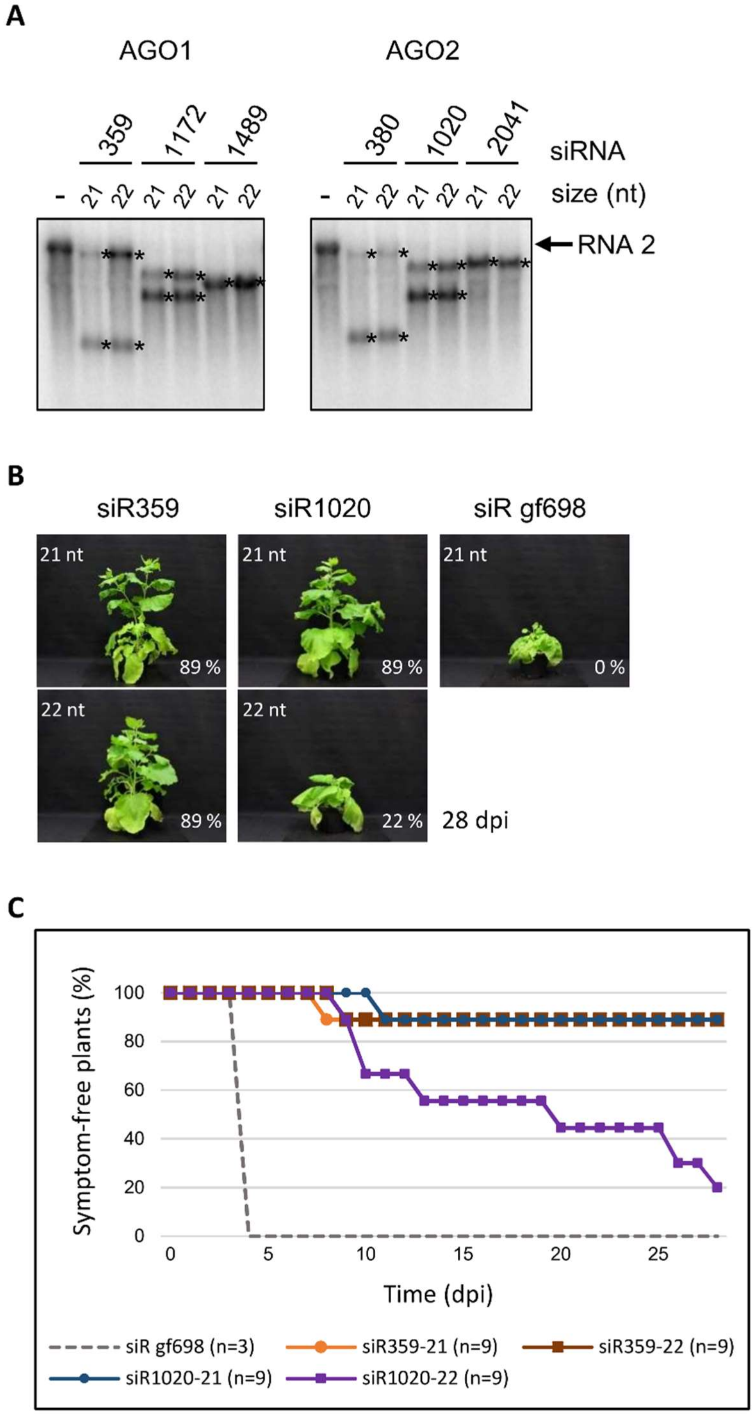

Supplementary Figure S4. Efficacy of 21 nt and 22 nt long *e*siRNAs against CMV RNA 2 *in vitro* and *in planta*. (A) Slicer assay using examples of 21 nt *e*siRNA as well as corresponding 22 nt variants (guide strands extended at the 3' end). **(B)** Comparison of the protective effect of 21 nt and 22 nt siRNA *in planta*. *N. benthamiana* plants were mechanically co-inoculated with the synthetic *e*siRNAs and with the genomic CMV RNAs (see text). Representative plant images 28 days after co-inoculation are shown. The percentage of asymptomatic remaining plants is indicated for each siRNA. **(C)** Percentage of

asymptomatic plants over the entire course of the experiment. The siRNA gf698 targeting GFP mRNA served as negative control. Results from one experiment including 9 plants for each CMV-targeting siRNA and 3 plants for the siR gf698 control.

Supplementary Figure S5

dsCMV6-21o
5'GGGCGAAUUGGGUACCGGGCAUUACGUUUCUUAAUUGCUGUAUACUCUUCUUAUGAUACGUUAUCA
GAUUUUUCAAGGUAAUCUUGAUGAGCUCCUUGUCGCUUUUUGUUCGAAGUUCUUACUCUUUCGUCG
AAAGUGCAGACUAU**UCUCAGAUCUGCCGGUCUCCC**UA3**'**

3'AU**CCCGCUUAACCCAUGGCCCGU**AAUGCAAAGAAUUAACGACAUAUGAGAAGAAUACUAUGCAAUAGU
CUAAAAAGUUCCAUUAGAACUACUCGAGGAACAGCGAAAAAACAAGCUUCAAGAAUGAGAAAGCAGCUU
UCACGUCUGAUA**AGAGUCUAGACGGCCAGAGGG5'**

dsCMV6-22o
5'GGGCGAAUUGGGUACCGGGCCAUUACGUUUCUUAAUUGCUGUAAUACUCUUCUUAUGAUACGUUAU
UCAGAUUUUUCAAGGUAAUCUUAUGAUGAGCUCCUUGUCGCUUUAUUUGUUCGAAGUUCUUACUCUG
UUCGUCGAAAGUGCAGACUAU**UCAUCAGAUCUGCCGGUCUCCC**UA3'

3'AU**CCCGCUUAACCCAUGGCCCGGU**AAUGCAAAGAAUUAACGACAUUAUGAGAAGAAUACUAUGCAAUA
AGUCUAAAAAGUUCCAUUAGAAUACUACUCGAGGAACAGCGAAAUAAACAAGCUUCAAGAAUGAGACAA
GCAGCUUUCACGUCUGAUA**AGUAGUCUAGACGGCCAGAGGG5'**

dsCMV
5'GGGCGAAUUGGGUACCGGGGAAUAACCGGGUACAUCGCGAGACGAGAUAUCUAUCUGAGCGUCGUCG
GCUUCACACUCUUCACUGUAGUAGAAAUCAGAUUUAGUGUA*AAUAGCCGCGACCAGGUCUUC*AAAACAC
UUCAUGGUUCG**GUUCAGAUCUGCCGGUCUCCCUA3'**

3'AU**CCCGCUUAACCCAUGGCCCCU**UAUUGGCCCAUGUAGCGCUCUGCUCUAUAGAUAGACUCGCAGCAG
CCGAAGUGUGAGAAGUGACAUCAUCUUUAGUCUAAAUCACAUUUAUCGGCGCUGGUCCAGAAGUUUUG
UGAAGUACCAAGC**CAAGUCUAGACGGCCAGAGGG5'**

dsGFP
5'GGGCGAAUUGGGUACCGGGCAAGAUCUGAGUCCGGACUUGUAuaguucauccaugccauguguAAUCCCAG
CAGCUGUUACAAACUCAAGAAGGACCAUGUGGUCUCUCUUUUCGUUGGGAUCUUUCGAAAGGGCAGAU
UGUGUGGA**CAUCAGAUCUGCCGGUCUCCC**UA3'

3'AU**CCCGCUUAACCCAUGGCCCGU**UCUAGACUCAGGCCUGAACAUAUCAAGUAGGUACGGUACACAUUA
GGGUCGUCGACAAUGUUUGAGUUCUUCCUGGUACACCAGAGAGAAAAGCAACCCUAGAAAGCUUUCCCG
UCUAACACACCU**GUAGUCUAGACGGCCAGAGGG5'**

Supplementary Figure S5. Structure and sequences of exemplary *e*dsRNAs and the control dsRNAs used (schematically shown in **Figure 4A)**. The sequences of the two strands of the respective dsRNAs are shown. The *e*dsRNAs contain six 21 nt or 22 nt long *e*siRNA sequences (guide strands indicated in different colors; see text and **Figure 4A**). The control RNA dsCMV contains a 126 nt long double-stranded fragment (corresponding to a length of six 21 nt long siRNAs) derived from CMV RNA 2. The fragment contains, by chance, two overlapping siRNAs that were identified as *e*siRNA candidates in the

screening procedure (siR557 and siR540; guide strand sequences underlined and italicized, respectively). The control RNA dsGFP contains a 126 nt-long double-stranded fragment of GFP mRNA including the sequence of the control siRNA siR gf698 used in previous experiments (guide strand sequence in lowercase). All dsRNAs contain pseudo-siRNA sequences at the ends (indicated in bold). The examples shown here generate a 2 nt 3’ overhang at both ends after annealing of the single-stranded components; however, *e*dsRNAs with blunt ends were also generated (see text). The different parts of the *e*dsRNAs dsCMV6-21o or -22o are indicated as follows (guide strand sequences each): Upper strand (5’-3’): bold black, pseudo-siRNA; red, siR1172; yellow, siR1489; brown, siR359. Lower strand (3’-5’): bold black, pseudo-siRNA; dark green, siR380; blue, siR2041; light green, siR1020 (see also **Figure 4A**).

Supplementary Figure S6

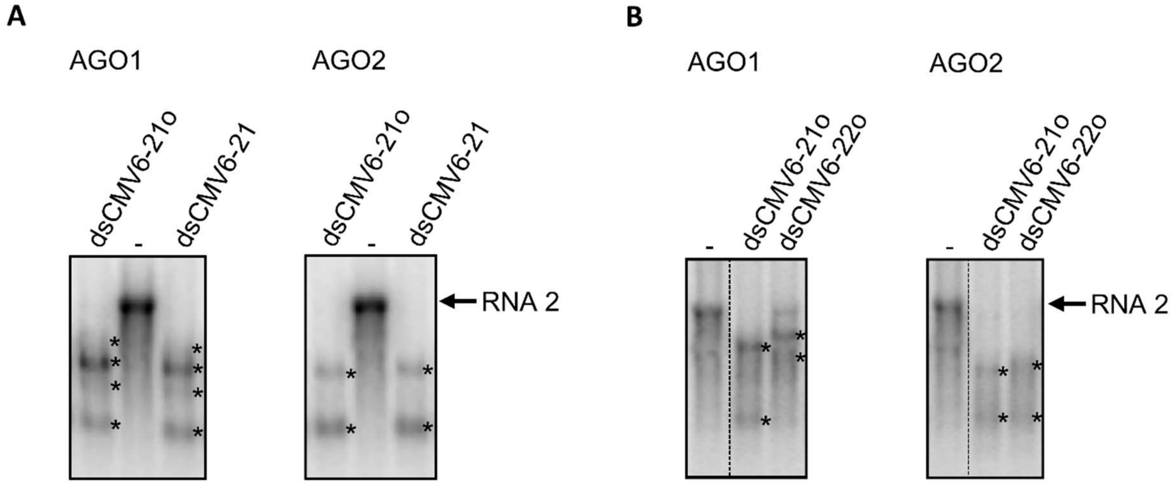

Supplementary Figure S6. Effect of termini structure and *e*siRNA size on the *e*dsRNA-mediated *in vitro* slicer activity. AGO1 or AGO2 mRNAs were translated in BYL in the presence of *e*dsRNAs that contained *e*siRNA sequences directed against CMV RNA 2. Thus, AGO1- or AGO2/RISCs were programmed with siRNAs that were processed from the *e*dsRNAs by DCLs present in BYL. ^{32}P-labeled, single-stranded CMV RNA 2 was added as a target, and siRNA-mediated cleavage was analyzed by denaturing agarose gel electrophoresis of total RNA and subsequent autoradiography. **(A)** Results of representative slicer assays performed with *e*dsRNAs having either blunt ends (dsCMV6-21) or 2 nt 3’ overhangs (dsCMV6-21o). The *e*dsRNAs contained six 21 nt *e*siRNAs (three active in experiments with AGO1, three active in experiments with AGO2). (**B**) Results of representative slicer assays performed with *e*dsRNAs containing either six 21 nt *e*siRNAs (dsCMV6-21o) or the six corresponding 22 nt *e*siRNAs (dsCMV6-22o). The latter were obtained by extending the guide strand of the 21 nt *e*siRNAs by one nucleotide. Asterisks (*) indicate the cleavage products.

Supplementary Figure S7

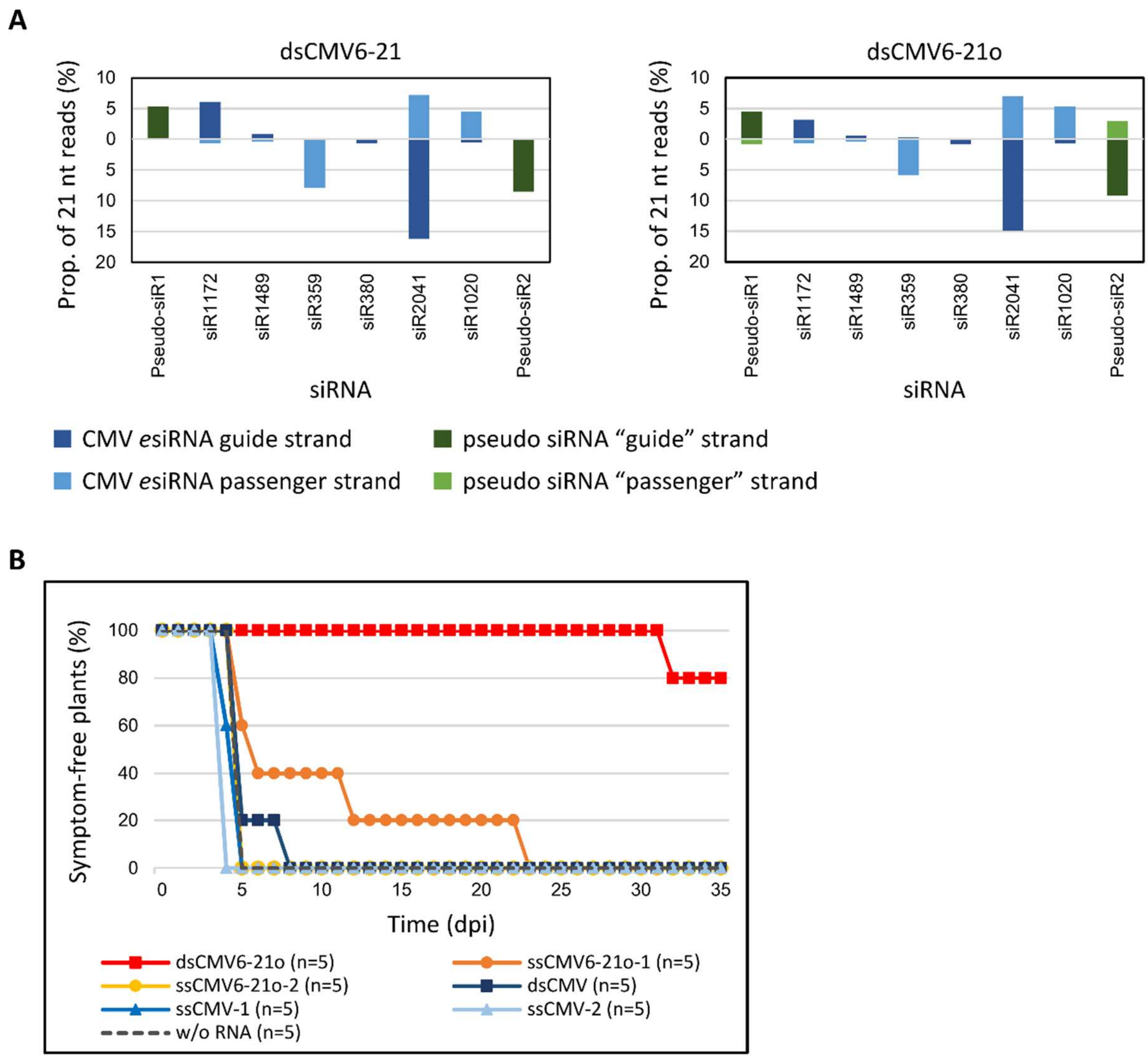

Supplementary Figure S7. RNA-seq analysis of siRNAs generated in BYL from *e*dsRNAs and comparison of the protective effect with single-stranded RNA. (A) dsCMV6-21 (blunt ends) and dsCMV6-21o (2 nt 3' overhangs) were processed in BYL by the endogenous DCLs. Total RNA was isolated, and the small RNA fraction analyzed by RNA-seq (see also **Figure 5**). The image shows the ratio of reads for the guide strand and the corresponding passenger strand for the six CMV RNA 2-targeting *e*siRNAs and the two pseudo-siRNAs on top of each other. In case of dsCMV6-21 there are no mapping 21 nt reads for the "passenger" strands of the pseudo-siRNAs, as the two 3' nucleotides are missing. (**B**) Comparison of the protective effect of double- and single-stranded RNAs *in planta*. *N. benthamiana* plants were mechanically co-inoculated with the genomic CMV RNAs and the *e*dsRNA dsCMV6-21o, the control RNA dsCMV, or the corresponding single-stranded RNAs (1 and 2) that make up the dsRNAs (see **Figure 4A** and **Supplementary Figure S5**). Inoculation with genomic CMV RNAs served as control. Plants were monitored for the appearance of CMV-specific symptoms for 35 dpi; the

image shows the percentage of asymptomatic plants over the entire period of the experiment. Results from one experiment including 5 plants per treatment are shown.

Supplementary Figure S8

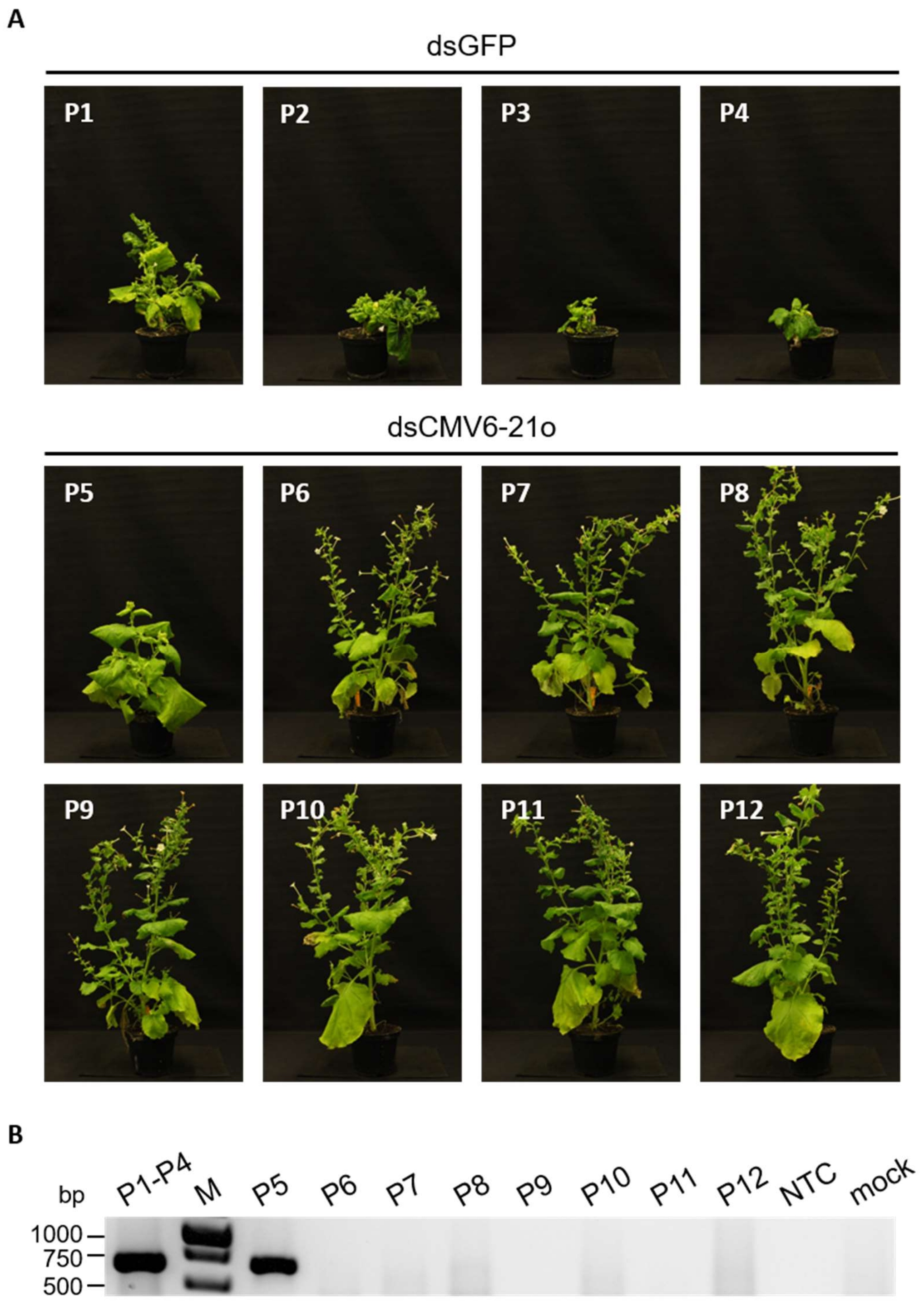

Supplementary Figure S8. Long-term protective effect of dsCMV6-21o against CMV infection. (A) Pictures of plants co-inoculated with dsGFP and genomic CMV RNAs (top panel) or dsCMV6-21o and genomic CMV RNAs (bottom panel) at 70 dpi. **(B)** CMV detection by RT-PCR. Leaf discs were collected

at 70 dpi. Total RNA was extracted and cDNA synthesized using Reverse Transcriptase (RT). PCR was performed to detect a 664 bp fragment of CMV RNA 2 and amplicons were separated on agarose gel. A pool of samples from symptomatic plants (P1-P4) co-inoculated with dsGPF and CMV served as positive control. A "no template control" (NTC, addition of water instead of cDNA during the PCR reaction) and RT-PCR over total RNA extracted from a plant inoculated with buffer (mock) served as negative controls (M = GeneRuler 1 kb DNA ladder).

Supplementary Figure S9

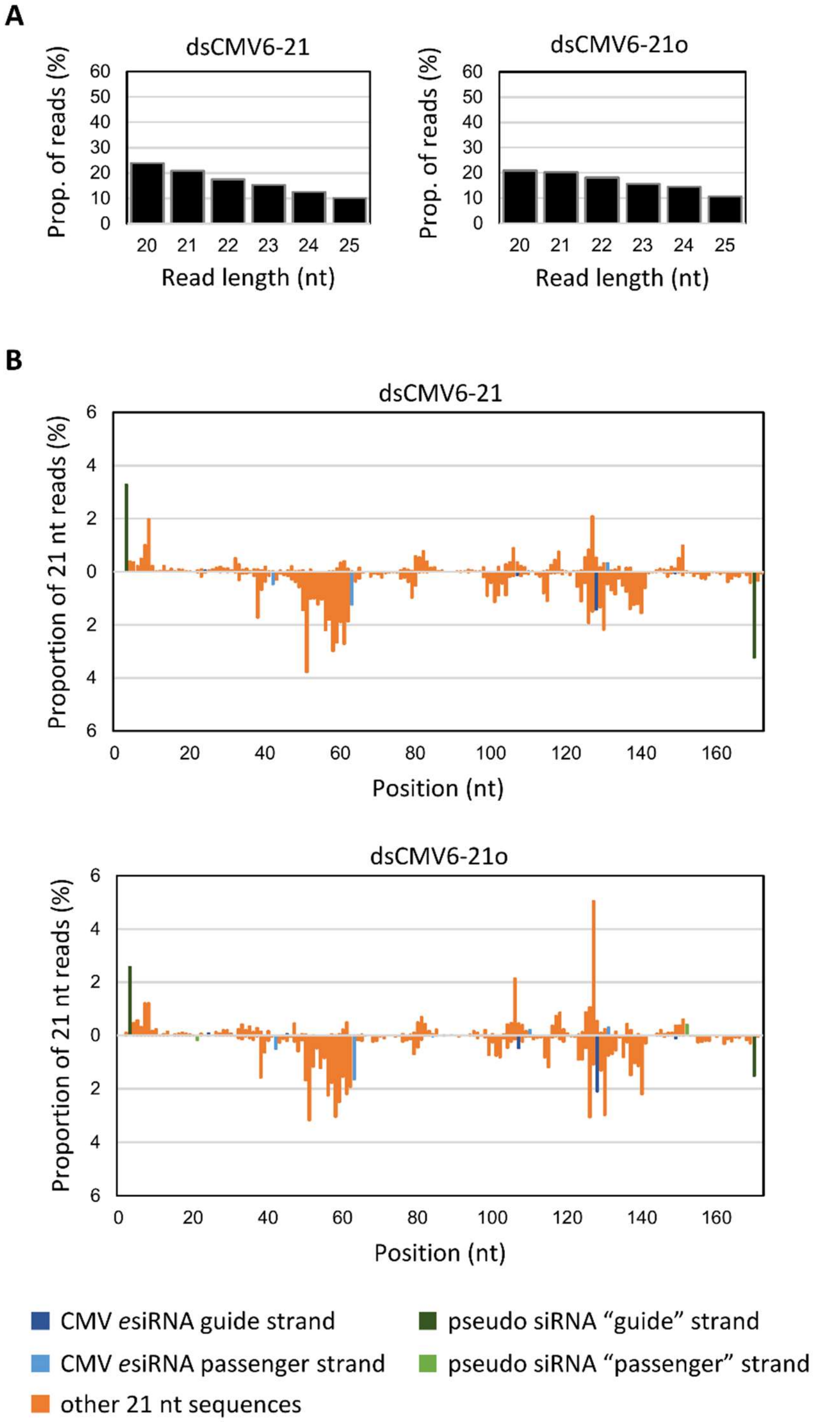

Supplementary Figure S9. Processing of *e*dsRNA *in planta*. dsCMV6-21 (blunt ends) and dsCMV6-21o (2 nt 3' overhangs) were rubbed onto *N. bethamiana* leaves in the same way as for the experiments demonstrating the protective effect of these *e*dsRNAs (see also **Figure 6**). Four hours after inoculation, total RNA was isolated from the treated leaf areas and the small RNA fraction analyzed by RNA-seq. (**A**) Size distribution of the 20-25 nt reads mapping to the *e*dsRNA sequences. (**B**) Proportion of guide and passenger strand reads among all 21 nt reads that mapped to the *e*dsRNA sequences; the peaks indicate the position of the 5' nucleotide of reads with respect to the *e*dsRNA. Peaks corresponding to the *e*dsRNAs contained pseudo-siRNA and *e*siRNA sequences are specifically colored (green and blue, respectively.

Supplementary References

1. Blum,H., Beier,H. and Gross,H.J. (1987) Improved silver staining of plant proteins, RNA and DNA in polyacrylamide gels. *Electrophoresis,* **8,** 93–99.
2. Bouché,N., Lauressergues,D., Gasciolli,V. and Vaucheret,H. (2006) An antagonistic function for Arabidopsis DCL2 in development and a new function for DCL4 in generating viral siRNAs. *EMBO J.,* **25,** 3347–3356.
3. Donaire,L., Wang,Y., Gonzalez-Ibeas,D., Mayer,K.F., Aranda,M.A. and Llave,C. (2009) Deep-sequencing of plant viral small RNAs reveals effective and widespread targeting of viral genomes. *Virology,* **392,** 203–214.
4. Wang,X.-B., Jovel,J., Udomporn,P., Wang,Y., Wu,Q., Li,W.-X., Gasciolli,V., Vaucheret,H. and Ding,S.-W. (2011) The 21-nucleotide, but not 22-nucleotide, viral secondary small interfering RNAs direct potent antiviral defense by two cooperative argonautes in *Arabidopsis thaliana*. *Plant Cell,* **23,** 1625–1638.
5. Qiu,Y., Wu,Y., Zhang,Y., Xu,W., Wang,C. and Zhu,S. (2018) Profiling of small RNAs derived from cucumber mosaic virus in infected *Nicotiana benthamiana* plants by deep sequencing. *Virus Res.,* **252,** 1–7.
6. Annacondia,M.L. and Martinez,G. (2021) Reprogramming of RNA silencing triggered by cucumber mosaic virus infection in Arabidopsis. *Genome Biol.,* **22,** 340.
7. Parent,J.-S., Bouteiller,N., Elmayan,T. and Vaucheret,H. (2015) Respective contributions of Arabidopsis DCL2 and DCL4 to RNA silencing. *Plant J.,* **81,** 223–232.
8. Katsarou,K., Mitta,E., Bardani,E., Oulas,A., Dadami,E. and Kalantidis,K. (2019) DCL-suppressed *Nicotiana benthamiana* plants: valuable tools in research and biotechnology. *Mol. Plant Pathol.,* **20,** 432–446.
